# Multimodal diagnostic strategies and precision medicine in mucinous ovarian carcinoma: a comprehensive approach

**DOI:** 10.3389/fonc.2024.1391910

**Published:** 2024-07-08

**Authors:** Yue Wang, Lina Peng, Wanlu Ye, Yanming Lu

**Affiliations:** ^1^ Department of Obstetrics and Gynecology, Shengjing Hospital of China Medical University, Shenyang, China; ^2^ Laboratory of Gynecologic Oncology, Department of Gynecology, Fujian Maternity and Child Health Hospital, Affiliated Hospital of Fujian Medical University, Fuzhou, China

**Keywords:** primary ovarian mucinous carcinoma, differential diagnosis, personalized precision medicine, tumor origin, multimodal diagnostic approach

## Abstract

Mucinous ovarian carcinoma (MOC) represents a distinct entity within ovarian malignancies, characterized by diagnostic challenges due to its rarity and the potential overlap with other tumor types. The determination of tumor origin is important for precise postsurgical treatment. This article highlights the accurate diagnosis and management of MOC, including the use of imaging modalities, serological tumor markers, immunohistochemistry, and genomic analyses. Transabdominal and transvaginal ultrasonography, complemented by MRI and CT, plays a pivotal role in differentiating MOC from other mucinous tumors and in surgical planning, particularly for fertility preservation. Serological markers like CA19-9, CA-125, and CEA, though not definitive, provide valuable preoperative insights. Immunohistochemistry aids in distinguishing primary MOC from metastatic mucinous carcinomas, while genomic profiling offers the potential for precision medicine through the identification of specific molecular signatures and treatment susceptibilities. Despite advancements in diagnostic techniques, no single method conclusively differentiates between primary and metastatic tumors intraoperatively. The paper reviews the origins, diagnosis, and differential diagnosis of primary mucinous ovarian carcinoma highlights the need for a multimodal diagnostic approach and advocates for the inclusion of MOC patients in clinical trials for personalized therapies, recognizing the heterogeneity of the disease at the molecular level.

## 1 Introduction

Recent studies have revealed a notable shift in the incidence rates of primary MOC. Historically, MOC accounted for an estimated 12% of all ovarian cancer diagnoses (1). Limited by histopathological knowledge, previous studies may have overestimated the prevalence of MOC. However, in the past three decades, with the update of MOC diagnostic criteria and the improvement of differential diagnosis, the incidence of MOC has declined. The latest data show that the prevalence of MOC is about 3%,with the prevalence of this condition is higher among young women aged 20 to 40 years, with no significant regional variations observed worldwide (2). This trend is not due to the real reduction of cases, but due to the improvement of diagnostic accuracy. Initially, all MOC cases were classified as primary, thereby inflating incidence statistics. Primary MOC was previously confused with benign, borderline and metastatic mucinous cancer (MMC) (3). Advancements in histopathological discernment have led to the reclassification of a significant number of cases previously diagnosed as MOC or mucinous ovarian borderline tumors to metastases from primary tumors of the gastrointestinal tract. Combined with an improved understanding of the biological features and clinical history of MOC, these advances have led to the recent recognition that the incidence of primary MOC is approximately 3% (2). This reevaluation underscores the importance of continual refinement in diagnostic methodologies to ensure accurate disease categorization and epidemiological understanding.

The 5th edition of the World Health Organization’s classification of ovarian neoplasms now delineates primary mucinous ovarian tumors into benign (mucinous cystadenoma and mucinous adenofibroma), borderline (mucinous borderline tumor), and malignant (MOC) categories, aligning with the categorizations for other epithelial tumors(e.g. serous ovarian tumors).

The treatment of MOC encounters numerous challenges. Presently, the diagnosis and differential diagnosis of MOC remain ambiguous, necessitating a reduction in misdiagnosis rates and the development of more precise diagnostic tools to enhance treatment outcomes. Due to the low incidence associated with MOC, there is an insufficiency of clinical trial data, resulting in the absence of comprehensive treatment guidelines. Furthermore, MOC exhibits low sensitivity to platinum-based and taxane chemotherapy drugs, conventional chemotherapy regimens for ovarian cancer have notable limitations when it comes to treating MOC. Currently, the options for targeted therapy in MOC are limited. Thus, identifying and developing specific targeted drugs is crucial for improving patient outcomes.

The clinical characterization, invasive behavior, prognosis, and therapeutic strategies for MOC have historically been subjects of debate, clouded by diagnostic inaccuracies. The site of tumor origin is critical to both treatment decision-making and prognostic determinations, as the efficacy of first-line treatments is contingent on targeting the primary tumor. This review aims to elucidate the origins, diagnosis, and differential diagnosis of primary MOC, with the goal of enhancing diagnostic precision and advancing the clinical management of patients afflicted with this complex condition.

## 2 Origins of mucinous ovarian tumors

Mucinous ovarian tumors (MOTs) are characterized by a dynamic and continuous evolutionary spectrum, with evidence suggesting a transitional continuum that spans from benign to borderline to malignant stages. These neoplasms are categorized under ovarian epithelial tumors; however, the intricacies of their histogenesis and precise cellular origins remain elusive. Multifactorial in nature, the proposed genesis of mucinous ovarian tumors encompasses a diverse range of sources. These include teratomas, mucinous metaplasia of ovarian surface epithelial cells or cortical inclusion body cysts and migrating cells at the tubo-peritoneal junction, Walthard nests/Brenner tumors, endometrioid tumors associated with endometriosis, and Mullerian mucinous tumors (4).

**Figure 1:**
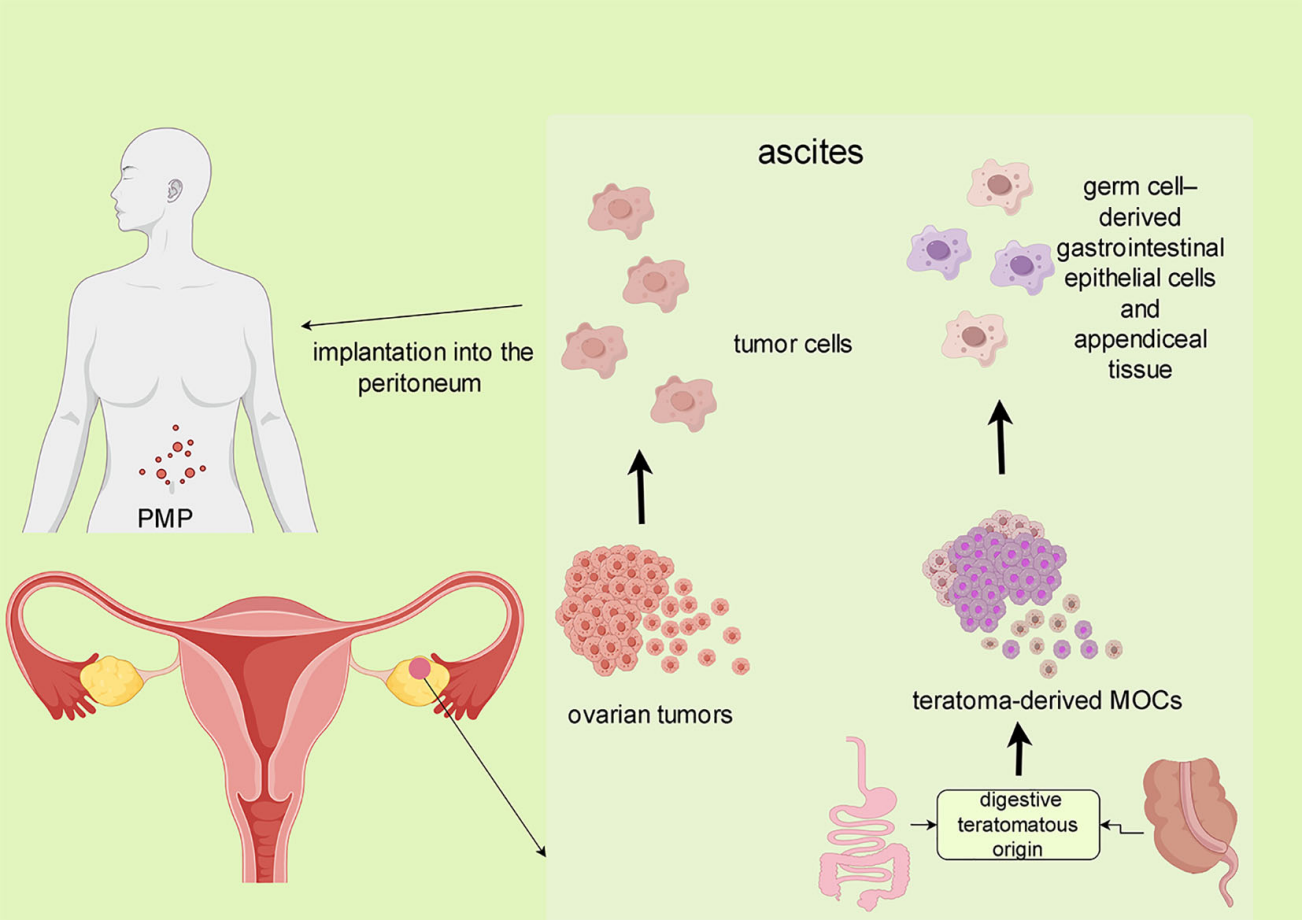
The formation of MOC-related PMP. The preoperative spontaneous or intraoperative rupture of the tumor and implantation into the peritoneum may lead to MOC-related.

Approximately 3–8% of mucinous ovarian tumors can be traced to teratomatous (germ-cell) origins, presenting with unique morphological and immunohistochemical characteristics, and other features (5). Tumors associated with ovarian teratomas exhibit a pronounced resemblance to gastrointestinal neoplasms, including intestinal (appendiceal) tumors, and share immunophenotypic features and morphological features consistent with upper gastrointestinal and pancreaticobiliary duct tumors. A significant study by Jung et al. (6) revealed that mature ovarian teratomas can evolve into mucin-secreting adenomas through the copy number amplification of chromosome 9’s short arm. This finding suggests that some mucinous ovarian tumors may develop from teratomas rather than being collision tumors (where two independent tumors coexist).Notably, pseudomyxoma peritonei (PMP), a condition characterized by the accumulation of mucinous material in the peritoneal cavity, is often associated with teratoma-derived mucinous tumors. In one study, PMP was observed in conjunction with 10 of the 42 teratoma-associated mucinous neoplasms (7). The occurrence of PMP in MOC may arise from spontaneous or surgical rupture and implanting into the peritoneum. Furthermore, the rupture of teratoma-derived MOCs containing malignant cells of gastrointestinal epithelial lineage could contribute to this phenomenon (8, 9). (**Figure 1**). As such, diligent evaluation for the presence of teratoma features is recommended in MOC cases presenting with PMP. Interestingly, patients with teratoma-associated mucinous tumors not involving the peritoneum generally have a more favorable prognosis compared to those with appendiceal/colorectal mucinous tumors affecting the ovary (7).

**Figure 2:**
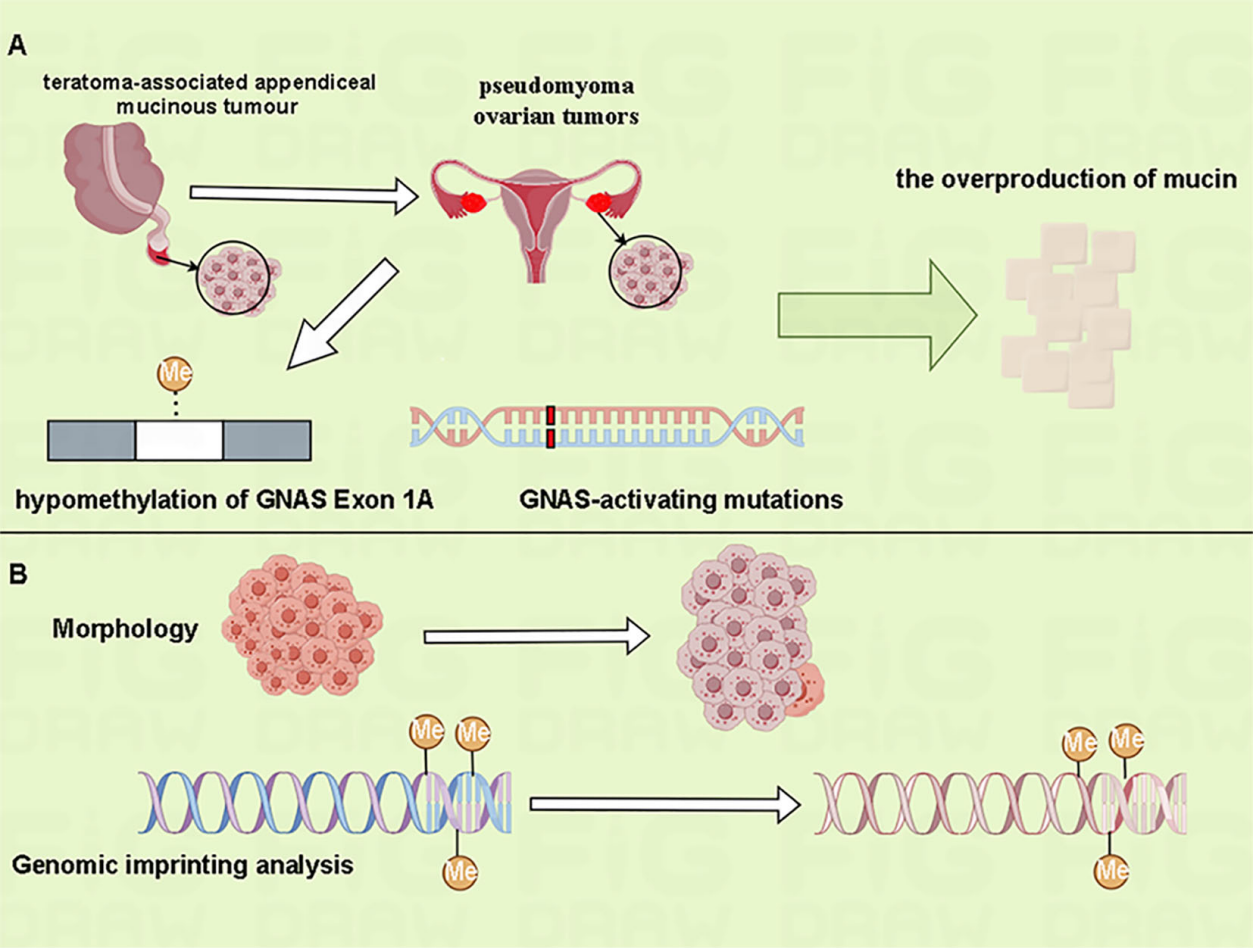
Genomic imprinting analysis can help determine the origin of mucinous ovarian tumors. **(A)** The aberrant methylation of GNAS exon 1A and GNAS-activating mutations may have been involved in the overproduction of mucin in teratoma-associated pseudomyxoma ovarian tumors. **(B)** The overall methylation characteristics of mucinous tumors and co-occurring teratomas are similar. Morphologically, however, the overgrowth of mucinous tumor can mask the original teratoma phenotype.

Kato et al. identified that aberrant methylation of the G-protein alpha-subunit (GNAS) exon 1A have been associated with pseudomyoma ovarian tumors in teratoma-associated mucinous tumors, and GNAS-activating mutations may contribute to mucin overproduction (10) (**Figure 2A**). Comparative methylation profiling indicates that the overall methylation profiles of mucinous tumors and co-occurring teratomas are usually similar, indicating a common origin from mature teratomas. In contrast, mucinous tumors without associated teratomas often present with somatic or irregular methylation signatures. Notably, the original teratoma tissue can be obscured in mucinous ovarian tumors that bearing teratoma-type methylation-imprinted genes, rendering the teratoma phenotype undetectable (10)(**Figure 2B**). Genomic imprinting studies provide a valuable tool for more accurately tracing the origins of these complex tumors.

Mucinous ovarian tumors of non-teratomatous origin typically exhibit an immunophenotype akin to that of the upper gastrointestinal tract, characterized by the expression of Cytokeratin 7 (CK7)-positive and Cytokeratin 20 (CK20)-positive or -negative (5). This immunophenotypic profile helps distinguish non-teratomatous MOTs from those deriving from teratomas. In general, non-teratomatous tumors usually present strong CK7 positivity with limited CK20 expression, whereas those arising from teratomas demonstrate a more varied profile, often showing CK7-negativity and CK20-positivity. An immunoprofile of CK7-negative, CK20-positive, and CDX2 Transcription Factor (CDX-2)–positive can suggest differentiation along the lower gastrointestinal tract lineage, though it is not definitive for a colorectal or appendiceal origin.

The condition known as synchronous mucinous metaplasia and neoplasia of the female genital tract (SMMN-FGT) manifests as a gastric-type multifocal disorder concurrently affecting multiple sites, such as the cervix, uterus, fallopian tubes, and ovaries. It is typified by coexisting cervical or endometrioid adenocarcinoma and MOC, and it must be differentiated from multiple primary malignant carcinomas (MPMCs) of the female genital system. Immunohistochemical studies have identified pronounced Mucin 6 protein (MUC6) expression in SMMN-FGT and have demonstrated that the Ki-67 Antigen (Ki-67) proliferation index is indicative of the malignancy’s aggressiveness (11). The intricate relationship between SMMN-FGT and mucinous tumors, including MOC and cervical adenocarcinoma, needs further scientific scrutiny.

## 3 MOC diagnosis and differential diagnosis

**Figure 3:**
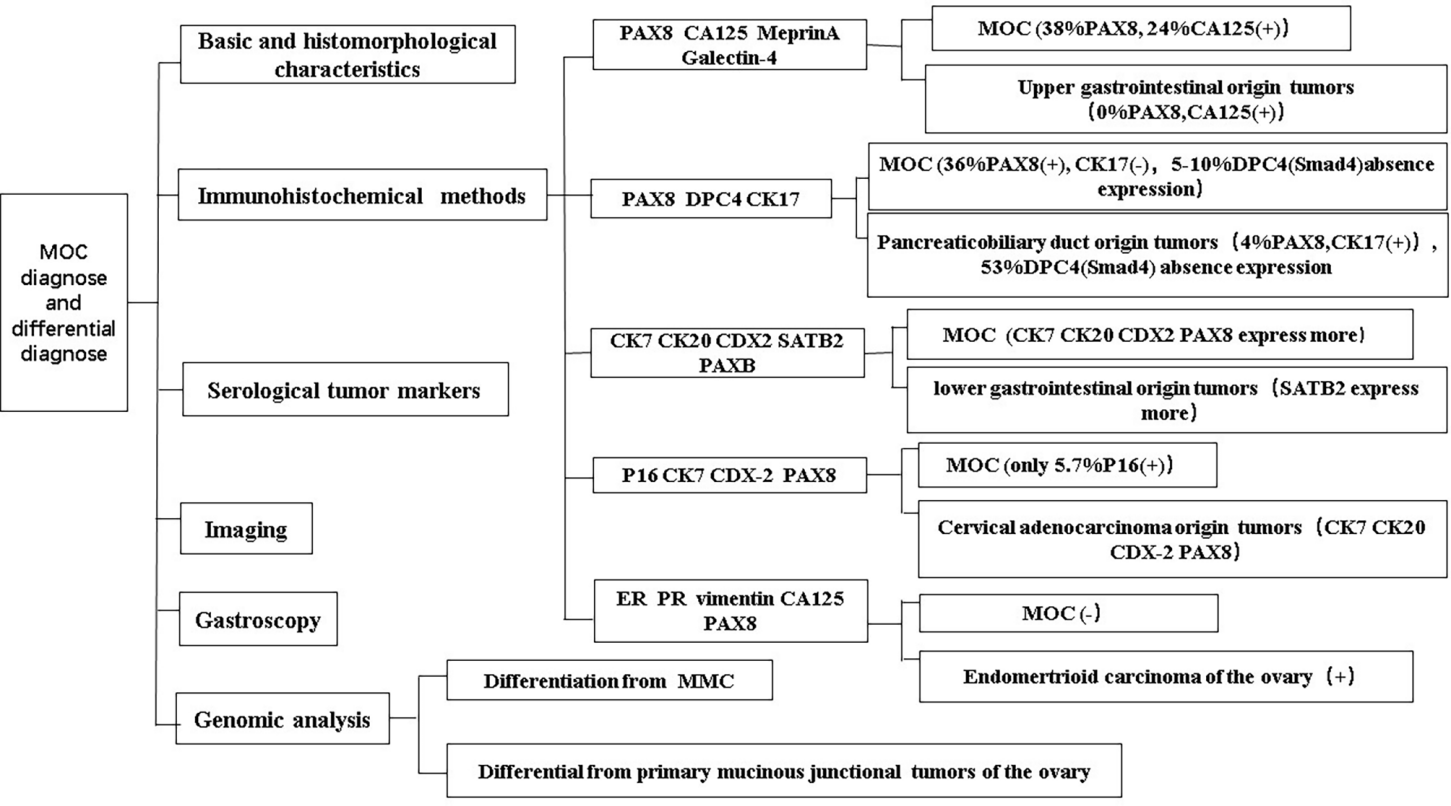
Diagnosis and differential diagnosis of MOC.

Accurately distinguishing primary MOC from metastatic ovarian tumors is of paramount importance in clinical practice, as it has significant implications for treatment strategies and patient prognosis. Despite this necessity, the current diagnostic methods face challenges in precisely differentiating between primary and metastatic ovarian tumors. This difficulty largely stems from the fact that the morphological features and immunophenotypic markers of primary MOC are not uniquely distinctive. For instance, certain primary MOC cases, particularly those arising from teratomas, exhibit morphological and immunohistochemical characteristics similar to intestinal and upper gastrointestinal tract tumors. It has been observed that low-grade malignant mucinous carcinomas predominantly show glandular differentiation of the intestinal type (approximately 80%), with a smaller proportion (around 20%) demonstrating intracervical type differentiation (12).In routine clinical practice, the initial approach to differentiating primary from metastatic MOC typically involves a combination of basic histomorphological analysis and immunohistochemical testing. These methods, while useful, may not always provide a definitive diagnosis, especially in complex or ambiguous cases. To further refine the differential diagnosis, particularly in the context of borderline mucinous tumors and ambiguous MOC cases, genomic analysis has emerged as a valuable tool (**Figure 3**).

### 3.1 Basic and histomorphological characteristics

Primary MOCs typically present as large, unilateral, and cystic masses, often with a multi-loculated structure, smooth external surfaces, and minimal nodularity. Age has been identified as a pertinent factor in distinguishing tumor origin, as demonstrated by one sample of 22 cases where 49% of primary MOCs occurred in patients under 50 years, in contrast to only 9.1% of metastatic cases (13). The dimensions of the tumor may also provide preliminary insight; median diameters for intersecting or malignant MOCs were reported at 18 cm (ranging from 3 to 40 cm), while benign counterparts averaged 10 cm (ranging from 2 to 60 cm) (14). Pathologically, MOC generally demonstrates an expansile growth pattern with infrequent and localized infiltrative changes. Pseudomyxoma peritonei (PMP) is highly specific to metastatic tumors. Moreover, the viscosity of the mucus in primary ovarian tumors is typically less dense compared to the gelatinous mucin characteristic of PMP arising from appendiceal malignancies (9). Low-grade mucinous appendiceal tumors metastasizing to the ovary often exhibit conspicuous multi-mucinous nodular changes (15). The detection of signet-ring cell within ovarian tissue is a highly specific indicator of metastatic origin, with a specificity of 99.7%, although sensitivity remains low at 12.0%, and the positive predictive value stands at 98.4% (16). Despite these indicators, relying solely on morphology to distinguish between primary and metastatic tumors can be challenging. Many tumors of malignant pancreaticobiliary origin, for example, closely resemble primary ovarian tumors and may present with features indicative of benign mucinous borderline tumors (17). Given the overlapping morphological, immunohistochemical, and even molecular features of primary and metastatic ovarian tumors, a thorough integration of clinical and pathological data is imperative for a definitive diagnosis.

### 3.2 Immunohistochemical analysis in MOC diagnosis

Immunohistochemical (IHC) profiling is indispensable for the precise pathological characterization of MOC. A case of combined ovarian and pancreatic tumors reported by Tucker et al. (18)exemplifies the critical role of IHC in differentiating primary from metastatic tumors, where initial imaging and histopathological analyses indicated an ovarian primary tumor; however, subsequent IHC and molecular studies revealed the pancreatic lesion as the primary source. In a comprehensive study by Dundr et al. (1), it was found that out of 14,060 cases of ovarian cancer, 656 (4.7%) were metastatic. The primary sources of these metastases varied, with colorectal cancer being the most prevalent (32%), followed by breast (15.4%), endometrial (12.9%), stomach (9.2%), appendiceal (6.7%), cervical (2.4%), pancreatic (2.2%), small bowel (1.6%), and gallbladder and biliary tract cancers (1.5%). Other cancers such as lung, skin, kidney, and esophageal were responsible for less than 1% each of the metastatic cases. Since the tumor tissue phenotype typically matches that of the originating cell line, and given that immunohistochemical markers bind specifically to these cell lines, tissue classification through immunohistochemistry provides a more reliable diagnostic approach compared to microscopic examination alone. Immunohistochemical analysis, therefore, enhances the diagnostic accuracy of MOC by enabling more precise differentiation between primary ovarian tumors and metastases. This accuracy is crucial for determining the appropriate treatment strategy, impacting the overall management and prognosis of the disease.

#### 3.2.1 Differentiation of MOC from gastric (upper gastrointestinal) origin tumors

MOC exhibits notable molecular and phenotypic similarities with upper gastrointestinal tumors, particularly adenocarcinomas at the gastroesophageal junction, in terms of histomorphology and molecular classification (19). The immunohistochemical expression profiles of CK7, CK20, CDX2, and Special AT-rich sequence-binding protein 2 (SATB2) bear striking resemblance between MOC and gastric-origin tumors. The differential expression of Paired Box Protein 8 (PAX8) and Carbohydrate Antigen 125 (CA125) aids in distinguishing MOC, with 35% and 24% of primary MOCs expressing these markers, respectively, a contrast to their absence in early gastric cancer (20). However, recent studies have shown that when mucinous gastric cancer metastasizes to the peritoneum, CA125 has a certain level of increase, so it is not easy to distinguish CA125 from MOC for mucinous gastric cancer that metastasizes to the peritoneum in the late stage (21, 22). In MOC, Estrogen Receptor (ER) and Progesterone Receptor (PR) are usually not expressed, and the expression in gastric cancer is related to the degree of cell differentiation and histological type. However, due to the low sensitivity of ER and PR, the diagnostic performance is not strong (23, 24).

Research by Heinzelmann-Schwarz et al. (25) has shown that in the tissues from 10 MOC and 38 gastrointestinal mucinous carcinoma cases, cytoplasmic galectin-4 displays consistent expression in both MOC and gastrointestinal mucinous carcinomas, albeit at significantly lower levels in MOC compared to gastrointestinal cancers. They also identified meprin A as a valuable adjunct marker for discerning primary ovarian mucinous adenocarcinomas from metastatic counterparts (25).Currently, CK7 and CK20 remain the standard immunohistochemical markers for ascertaining the tissue origin of mucinous ovarian adenocarcinomas.

#### 3.2.2 Discrimination of MOC from pancreatobiliary duct-origin tumors

Differentiating primary MOC from metastatic tumors originating from the pancreaticobiliary tract is a complex process, primarily due to the overlapping marker positivity rates between these two tumor types.PAX8, Deleted in Pancreatic Carcinoma 4 (DPC4), also known as Smad4, and Cytokeratin 17 (CK17) serve as critical differential markers. PAX8 is present in 36% of primary MOC cases, compared to a minimal 4% in pancreatobiliary malignancies (1). The lack of DPC4 expression, which occurs in 53% of pancreatic cancers and 5–10% of primary MOCs, is a significant diagnostic feature suggestive of pancreatobiliary tract metastasis (26, 27). Such absence is considered to be a landmark differential point for the confirmation of MMC of pancreaticobiliary origin (18). Meanwhile, CK17 is typically absent in mucinous ovarian tumors but is found in 27–83% of metastatic pancreatic cancers, marking its expression as an indicator of pancreatic origin (28). The study by Yang et al. (29) revealed that claudin-18 is a sensitive and specific marker for adenocarcinomas arising from the gastric and pancreaticobiliary ducts, boasting a sensitivity of 79% and a specificity of 93%. However, claudin-18 positivity was also noted in over half (more than 54%) of MOC cases, indicating that while useful, claudin-18 expression alone cannot definitively distinguish MOC from tumors of pancreaticobiliary duct origin. Mucin 6 (MUC6) is primarily expressed in the upper gastrointestinal tract and pancreas. In primary MOC, MUC6 expression is typically low. In contrast, mucinous carcinomas originating from the upper gastrointestinal tract exhibit high MUC6 expression, with a positivity rate of 76% (31/41 cases) (30).

#### 3.2.3 Distinguishing MOC from lower gastrointestinal-origin tumors

The diagnostic distinction of primary MOC from lower gastrointestinal metastases (colorectal and appendiceal cancers) relies on the nuanced interpretation of immunohistochemical markers including CK7, CK20, CDX2, SATB2, and PAX8. CK7 and CK20 are crucial markers in this context. CK20, a marker with high tissue specificity, is naturally present in the gastrointestinal mucosa and is commonly upregulated in gastrointestinal tumors. CK7, although more broadly distributed, is typically absent in colorectal cancers and is present in approximately 90% of primary MOC cases, often diffusely expressed in approximately 85% of the tumor cells (1). In metastatic from colorectal and appendiceal cancers, CK7 is detected in a minority of cases—31% and 26% respectively—with only 6% and 13% showing diffuse expression (1). CK20 is detected in 65–70% of primary MOC cases and is diffusely present in around 40% of these cases. However, CK20 is detected in the 90% and 92% colorectal and appendiceal metastases, with high rates of diffuse expression (1). In terms of co-expression, primary MOC demonstrates CK7+/CK20+ in 67% of cases, CK7+/CK20- in 26% and CK7– CK20+ positive in 7% of cases. Interestingly, MOC of teratomatous origin often shows a CK7-/CK20+ profile in 50% of the cases (1). In comparison, only a fraction of appendiceal and colorectal cancer cases exhibit both markers (22% and 11%, respectively), 78% and 79% of these cases, respectively, are CK7– CK20+, 0% and 3% are CK7+ CK20–, and 0% and 6% are CK7– CK20– (1).

CDX2, a transcription factor crucial in gastrointestinal development, manifests in 49% of primary MOC cases with strong expression in 26%. However, its strong expression is much more prevalent in colorectal (93%) and appendiceal (97%) cancers (1). SATB2, another marker with high specificity for colorectal and appendiceal malignancies (31), is typically absent in primary MOC but diffusely expressed in teratoma-associated MOC (9). Meagher et al. (24) have established that a lack of CK7 coupled with any level of SATB2 expression is indicative of a primary gastrointestinal tumor. focal CK7 positivity with diffuse SATB2 positivity also reflects a primary gastrointestinal tumor, Conversely, diffuse CK7 expression with absent or negative SATB2 expression points to primary ovarian cancer, while diffuse positivity for both CK7 and SATB2 suggests a lower gastrointestinal origin. PAX8, noted for its limited expression in ovarian tissue, is weakly and focally expressed in about 35% of MOC cases, contrasting with its 5% expression in appendiceal cancers and absence in colorectal cancers (1).

IMP3 has been observed to vary significantly between metastatic and primary mucinous ovarian adenocarcinomas, with its expression correlating with tumor aggressiveness (32). While typically negative or weak positive in primary ovarian lesions, IMP3 tends to be moderate to strong in metastatic lesions, especially those affecting the uterus and greater momentum (32). The diagnostic potential of IMP3 in differentiating primary from metastatic MOC needs further investigation.

Mucin2 (MUC2) is a secretory mucin predominantly expressed in the lower gastrointestinal tract. In metastatic colorectal adenocarcinoma involving the ovary, MUC2 has an expression rate of approximately 51%, whereas it is not expressed in MOC (33). Conversely, Mucin5AC (MUC5AC) is almost not expressed in colorectal-origin mucinous carcinomas, but shows diffuse positive expression in primary MOC, with an expression rate of 86% (34).

A multi-marker immunohistochemical approach significantly enhances the accuracy of distinguishing primary from metastatic MOC. The concomitant assessment of CDX2, CK7, and Dipeptidase 1 (DPEP1) has demonstrated high diagnostic precision—97% for primary MOC (16/16 tumors) and 100% for metastatic rectal cancer (16/16 tumors). However, the accuracy for detecting upper gastrointestinal metastases remains lower at 56% (9/16 cases) (35). Given the distinct immunophenotype of teratoma-derived MOC, reliance solely on immunohistochemical results is inadequate, and genetic testing is recommended to provide a comprehensive diagnostic conclusion.

#### 3.2.4 Distinguishing MOC from cervical adenocarcinoma

Tumors associated with human papillomavirus (HPV) of cervical adenocarcinomas origin, can present with histological similarities to primary MOC (36). However, the expression of the cell cycle regulatory protein p16 provides a valuable discriminative marker. Primary MOC is only diffusely positive for p16 in a minor fraction of cases—approximately 5.7% (37). In contrast, p16 has been identified as a highly sensitive (100%) and specific (98%) marker for the identification of ovarian metastases stemming from cervical adenocarcinoma (38). Non-HPV-associated cervical adenocarcinomas of gastrointestinal origin usually express CK7, with about 50% of these tumors also expressing CK20 and CDX-2. Typically, ER and PR are not expressed, while PAX8 may be positive in these cervical tumors. They may also exhibit limited positivity for gastric mucin differentiation markers, such as MUC6 and the Anti-Mucin antibody1083 (HIK1083), though these markers lack sensitivity and specificity for this tumor type (39).

#### 3.2.5 Distinguishing primary MOC from endometrioid ovarian carcinoma

Endometrioid ovarian carcinoma typically exhibits positive immunoreactivity for ER and PR, as well as for CA125 and vimentin, markers that are generally negative in primary MOC. Notably, with the confirmation of endometrial histology based on PR and waveform protein expression (40), approximately one-fifth (19.6%) of cases previously classified as MOC have been reclassified as endometrioid carcinomas (41). The expression of PAX8, a transcription factor, further aids in the distinction between these two entities. While endometrioid carcinoma commonly shows diffuse and strong PAX8 positivity, primary MOC is often negative or only weakly positive for this marker (24). The work of Wookbeck et al. (40) emphasizes the diagnostic precision of using a combined immunohistochemical profile of PR and vimentin. This approach has been reported to accurately differentiate ovarian endometrioid carcinoma from MOC with over 95% accuracy, underscoring the utility of a multimarker strategy in refining ovarian cancer subtyping.

#### 3.2.6 Summary of diagnostic approaches

The therapeutic management of primary versus metastatic ovarian tumors differs markedly, yet intraoperative challenges often impede definitive diagnosis. Limitations in rapid intraoperative pathology sampling for frozen pathological analysis and the inability to perform timely immunohistochemistry present significant obstacles. Consequently, the most efficacious approach for intraoperative diagnosis leverages a combination of macroscopic tumor assessment, histological examination, and careful review of the patient’s clinical history. Definitive pathological conclusions drawn from paraffin-embedded specimens are crucial for guiding subsequent treatment strategies.

Accurate differentiation is imperative, especially in distinguishing MOC from conditions with similar presentations. High-grade serous carcinoma (HGSC), more prevalent than MOC, is typically distinguishable through gross morphological inspection and immunohistochemical markers such as Wilms Tumor 1 protein (WT1) (42). A comprehensive summary of immunohistochemical markers employed in the identification of MOC and its differentiation from other tumor origins, such as MMC and HGSC, is detailed in **Table 1**.

**Table 1:** Immunohistochemical identification of MOC with different sources MMC and HGSC (15, 31, 32, 40, 43, 44).

Immune-histochemistry	HGSC	MOC^a^		MMC					
		Intestinal Type	Endocervical Type	Appendix	CRC^b^	Pancreatic	Gastric	Cervical	Endometrium
SATB2	None	- ^d^	–	+ ^c^	+	–	–	None	-/+^f^
CDX2	–	+/-^e^	–	+	+	+/-	+/-	-/+	+
CK7	+	+	+	–	–	+/-	+/-	+	None
CK20	–	+/-	–	+	+	-/+	-/+	-/+	None
MUC6	None	+/-		None	None	+/-	None	+	None
DPC4	+	+	+	+	+	+or-	+	+	None
CEA	–	+/-	–	+	+	+/-	+/-	+/-	None
CA19-9	–	+	-/+	None	+	+	+	–	None
CA12-5	+	–	+	–	–	+/-	–	None	+
PAX8	None	-/+		+	–	+/-	–	+	+
ER	+	–	+	–	–	–	–	-/+	+
PR	+	None	None	–	–	–	–	None	+
P16	–	–	–	None	-/+	–	–	+	None

^a^MOC, Mucinous Ovarian carcinoma; ^b^CRC, Colorectal carcinoma;

^c^+:diffusely; ^d^ -:diffusely negative; ^e^+/-:diffusely positive or focally negative; ^f^-/+:diffusely negative or focally positive.

### 3.3 Genomic analysis

Innovations in genomic technology now allow for the sophisticated analysis of tumors that present synchronously at multiple sites. By elucidating the homology of these neoplasms, clinicians can ascertain whether they represent independent primary tumors or metastatic spread from a single origin. This genomic insight is crucial for tailoring personalized postoperative treatment regimens, marking a significant leap forward in precision oncology.

#### 3.3.1 Genomic distinction between MOC and MMC

MOC is genetically distinct from other subtypes of epithelial ovarian cancer and shares several molecular characteristics with gastrointestinal tract tumors. The prevalence of Kirsten rat sarcoma viral oncogene (KRAS) mutations in MOC is slightly higher compared to mucinous and non-mucinous colorectal carcinomas, with mutations observed in 43–46% of MOC cases versus 30% of mucinous colorectal tumors. Furthermore, approximately 20% of mucinous colorectal carcinomas exhibit mutations in V-raf murine sarcoma viral oncogene homolog B1 (BRAF) (42).The amplification of Human Epidermal Growth Factor Receptor-2 (HER2), also known as ERBB2, is found in approximately 20–30% of invasive MOC cases and 6% of mucinous borderline tumors (MBTs), contrasting with its rarity in colorectal and non-mucinous carcinomas (42). Conversely, Tumor Protein P53 (TP53) mutations are emblematic of high-grade serous carcinoma (HGSC), identified in 96% of HGSC cases but less than 30% of MOC (43). Ohnishi et al. (45) discovered KRAS mutations in 43.8% of MOC cases (7/16cases) and 20% of MBTs(2/10cases), yet found no such mutations in benign mucinous ovarian tumors. BRAF mutations were present in four MBTs but absent in MOC and benign tumors. Notably, TP53 mutations were not observed in their study (45). These findings suggest a possible association between KRAS mutations in MBTs and the progression to invasive MOC, while BRAF mutations do not exhibit a similar correlation.

#### 3.3.2 Genomic distinctions between primary MOC and mucinous ovarian borderline tumors

Genomic studies reveal significant mutational overlap among benign, borderlin, and malignant mucinous ovarian tumors. Cheasley et al. (46) identified copy number alterations as pivotal in the malignant transformation and metastatic potential of mucinous ovarian tumors, suggesting their value as prognostic indicators. Benign mucinous ovarian tumors frequently harbor mutations in KRAS or Cyclin-dependent kinase inhibitor 2A (CDKN2A). Mucinous borderline tumors, which often develop in the presence of these mutations, may also exhibit additional genomic copy number variations. A high prevalence of CDKN2A mutations has been documented in both MOC (91%) and mucinous borderline tumors (95%) (46). Concordantly, other research has correlated KRAS and CDKN2A pathway alterations withmucinous cystadenomas and borderline tumors (47). CDKN2A and KRAS occur in the early stages of tumor development (42). Common genomic features shared by benign, borderline, and malignant mucinous ovarian tumors include the overall mutational burden and the number of point mutations.TP53 pathogenic variants, which increase significantly in malignancy, suggest a driving role in the malignant evolution of these tumors (46, 48). The transition from borderline to malignant status is often marked by TP53 mutations or amplifications (49). Notably, the frequency of KRAS mutations increased from benign to borderline to malignant stages, underscoring its importance in the continuum of cancer developmen (50). In another study, BRAF mutations, while rarer, appear more frequently in malignant carcinomas compared to their benign and borderline counterparts (51).The intricate molecular landscape, detailing the mutational profiles of MOC, mucinous borderline tumors, HGSC, and colorectal cancer, is systematically presented in **Table 2**.

**Table 2:** Molecular mutation profiles in MOC, BMOT, HGSC and CRC (31, 40, 43, 45).

Molecular mutation	MOC	BMOT	HGSC	CRC
KRAS	33-46%	20%	10-22%	31-48%
BRAF	0-9%	40%	None	15-27%
TP53	26-55%	None	99%	31-41%
HER2	18-35%	6%	None	<1%
MSI-H	22%	None	13.80%	25-36%
APC/CTNNB1	9%	None	None	24%
BRCA	None	None	50%	None

### 3.4 Advances in imaging for MOC diagnosis

Transabdominal and transvaginal ultrasonography remain cornerstone imaging techniques in the evaluation of gynecological conditions. In recent years, ultrasonography has become increasingly instrumental in differentiating between benign and malignant ovarian tumors. On ultrasound, mucinous tumors often appear larger than serous tumors and up to half of mucinous tumors exhibit internal septations, whereas serous tumors more commonly present as solid masses or with papillary structures (52).Given the often sizable dimensions of mucinous tumors, comprehensive imaging assessment typically necessitates the incorporation of computed tomography (CT) or magnetic resonance imaging (MRI) alongside ultrasound. Contrast-enhanced CT is the modality of choice in clinical settings for identifying primary tumors, determining the extent of tumor spread for staging purposes, and detecting complications associated with advanced ovarian cancer, such as bowel obstruction. However, its utility in characterizing the tumor type is limited. MRI is particularly valuable in differentiating malignant mucinous ovarian tumors from benign and borderline counterparts (53, 54). It also provides a clearer distinction between MOC and metastatic mucinous carcinomas, thus playing a crucial role in informing surgical planning and treatment strategies (52).

MRI offers superior soft-tissue contrast compared to CT, providing a distinct advantage in the imaging of ovarian tumors. The diverse mucinous and protein content of plasmacytotic and mucinous ovarian tumors imparts distinct T1 and T2 signal characteristics on MRI. Mucinous tumors frequently exhibit a “stained glass” appearance due to their heterogeneous content. In contrast, serous tumors, often referred to as plasmacytomas, tend to be bilateral and unicystic, with gritty calcifications detectable upon histological examination. A higher signal intensity on T1-weighted images usually correlates with a higher mucin concentration within the cystic fluid. Malignant MOCs typically demonstrate pronounced enhancement on MRI, attributable to the rapid proliferation of tumor cells. This results in thicker cystic walls, more pronounced internal septa, and an increase in papillary structures, solid components, and compartments. Additionally, the vascular perfusion within these tumors is often more extensive, which, when combined with lower apparent diffusion coefficients (ADCs), distinguishes them from MBTs. ADC values, which are derived from diffusion-weighted imaging (DWI), tend to decrease as the malignancy of ovarian tumors increases. This reflects the reduced extracellular space, increased cellularity, and heightened proliferative activity within the tumor (54). DWI has become a pivotal tool in differentiating benign, borderline, and malignant ovarian tumors, with a sensitivity ranging between 83.3% and 93.1% for distinguishing between benign and malignant forms (53). DWI also exhibits a high sensitivity in detecting peritoneal metastases (55).

MRI has emerged as a critical tool in the distinction between MBTs and MOCs. In a comparative analysis of MRI data from 75 MBI and 38 MOC cases, Yang et al. (56) discerned that while MBTs typically present as predominantly cystic, MOCs are often cystic with significant solid components and tend to have lobulated or irregular shapes with indistinct borders, whereas MBTs were regular in shape. Papillary nodules, a feature observed in both MBTs and MOCs, are more prevalent and pronounced in MOCs. These nodules appear small and hypointense on T2-weighted MR images in MBTs, in contrast to their appearance in MOCs (57). The majority (59.2%) of patients with MBTs had only small amounts of physiological ascites, whereas 71.8% of those with MOC had moderate to large amounts of ascites (56). Yang et al. (56) identified that the presence of papillary nodules, tumor size, degree of enhancement, solid component ratio, and ADC values associated with ascites are independent MRI features that enhance the differentiation between MBTs and MOCs,with an area under the curve of 0.949, sensitivity of 82.1%, and specificity of 97.4%. MRI also assists in pinpointing the primary site of the tumor. MMCs of gastric and breast origins usually present as substantive solid masses, with gastric metastases often resembling Krukenberg tumors, which have the macroscopic features of lobulated solid tumors. Breast cancer metastases are usually small (<5 cm), with characteristic multinodular or polyp-like protrusions. By contrast, metastases from the appendix, colorectum, and pancreaticobiliary system are more cystic, posing a diagnostic challenge due to their resemblance to MOC (55, 58). MOC typically exhibits as a multifocal cystic lesion with an intermediate-intensity solid component on T2-weighted images, with a high signal on DWI and a –3 enhancement curve (i.e., earlier enhancement relative to the myometrial curve) on perfusion sequences. For MMC, T2-weighted MRI shows the internal structure of metastases, which usually have heterogeneous T2 signal intensity due to varying degrees of cystic degeneration. Ovarian metastases of rectal cancer can be classified into four types according to their imaging and general presentations, and the fourth type is most similar to MOC. Depending on its solidity of composition, number, and location, rectal-derived MMC appears as a typical multi-compartmental cystic lesion with a “stained glass” appearance on T1-weighted MRI, and has a hyperenhanced necrotic component on T2-weighted MRI. Gastric-derived MMC does not have a distinctive MRI appearance and has more solid and fewer cystic components than does enteric-derived MMC (57). Thus, the MRI findings enabling the differentiation of primary mucinous ovarian tumors from other entities are high T1 and low T2 signal intensity, wall nodules, and solid components.

CT complements MRI in tumor staging and is particularly useful for identifying gastrointestinal involvement, crucial for ruling out appendiceal tumor (59). While Positron Emission Tomography CT (PET-CT) provides a more accurate determination of tumor origin and stage, it is less accessible due to cost constraints. Hence, enhanced CT and MRI are recommended as more feasible options for patients unable to undergo positron emission tomography CT.

In summary, CT is instrumental in tumor staging, whereas MRI provides a more nuanced differentiation between benign and malignant tumors, assisting in prognostication. In addition to the identification of tumor characteristics (e.g., papillae, cystic wall), images should be reviewed in detail to identify involvement of the gastrointestinal tract (i.e., appendix, colon, and/or stomach), lymph nodes, and peritoneum. Preoperative imaging exploration significantly contributes to reducing misdiagnosis and preventing unnecessary surgical interventions.

### 3.5 Gastroenteroscopy

Gastroenteroscopy stands as a pivotal diagnostic procedure in distinguishing primary ovarian tumors from those of gastrointestinal origin. This endoscopic examination becomes particularly vital when imaging studies indicate a non-ovarian source or when the tumor is at an advanced stage. Additionally, a serological marker ratio—specifically, CA125 (U/ml) to Carcinoembryonic antigen (CEA) (ng/ml) of 25 or less—warrants gastroenteroscopy to further investigate a possible gastrointestinal etiology (59). Definitive diagnosis of MOC often necessitates a thorough exclusion of gastrointestinal primaries and related malignancies. MOC can be diagnosed only after the exclusion of gastrointestinal and homologous tumors when gastrointestinal endoscopic or angiographic findings are positive.

### 3.6 Serological tumor markers

Serological tumor markers, including Carbohydrate Antigen 199 (CA19-9), CA-125, and CEA, are crucial in the preoperative assessment of mucinous ovarian tumors and provide valuable insights during the evaluation of frozen sections. CA-125 is recognized as a specific marker for ovarian malignancies, yet it often manifests at lower levels in MOC. In contrast, CA-19-9 shows a more robust association with mucinous ovarian tumors compared to other epithelial ovarian malignancies. The diagnostic sensitivity and specificity of CA19-9 for distinguishing between borderline (formerly borderline tumor) and malignant mucinous tumors are 52.7% and 83.8%, respectively. For CA-125, these figures are 68.2% for sensitivity and 83.9% for specificity. CEA’s sensitivity and specificity stand at 31.9% and 90.8%, respectively (14). Ohya et al. (60) suggest that serum CEA levels exceeding twice the threshold value can be used to differentiate malignant from benign tumors, whereas CA-19-9 levels above the cut-off could be used to identify borderline tumors.

## 4 Discussion

The diagnostic refinement of MOC poses a significant challenge due to its rarity and the potential for misdiagnosis as other malignancies. Accurate initial tumor identification is crucial to avoid unnecessary surgical interventions; while surgery remains the cornerstone of MOC management, MMCs are typically addressed with radiotherapy. The preoperative characterization of mucinous ovarian tumors, including grading are instrumental in surgical planning. These assessments not only inform surgical strategy to minimize recurrence but are also pivotal in preserving fertility in women of childbearing potential. Preoperative imaging (i.e., MRI) examination and the analysis of serum tumor markers are of great value for these purposes. Immunohistochemistry and genomic analyses hold promise for enhancing the identification accuracy of primary MOC. However, the search for MOC-specific molecular signatures demands further research. Currently, the use of immunohistochemical markers is necessary for differential diagnosis, but multiple markers should be detected jointly to determine tumor origin. And combined phenotypic and genotypic insights underpin the application of precision medicine. Genomic profiling offers detailed insights that can guide differential diagnosis and subsequent MOC treatment strategies. While genetic testing can inform individualized treatment plans, more extensive data is required to support this personalized approach fully. Despite the availability of these numerous methods, no method currently enables the definitive differentiation of metastatic and primary ovarian tumors, especially with regard to the intraoperative determination of tumor origin and borderline or malignant nature. Occasionally, the origin of certain tumors remains elusive despite exhaustive investigation. MOC may evolve from MBTs, and such progression may necessitate additional surgery. Genetic evaluation of MBTs could predict disease evolution and inform prognostication, embodying the principles of precision medicine. Advanced MOC exhibits limited responsiveness to conventional platinum-based chemotherapy regimens, which are the standard for gynecological malignancies. Current cutting-edge research emphasizes the heterogeneity of tumors at the tissue and molecular levels, leading to the argument that a tumor does not occur as a single disease, but rather as a group of distinct tissue subtypes with important differences in genetics, morphology, tumorigenesis, prognosis, chemotherapy sensitivity, and, in particular, molecular features that may serve as new target (12). This diversity necessitates a reconsideration of MOC as not merely a single disease but rather a spectrum of molecularly distinct entities. Consequently, integrating MOC patients into trials for experimental therapies—tailored to specific molecular profiles—may prove more efficacious than the application of generic gastrointestinal chemotherapy protocols.

## Author contributions

YW: Conceptualization, Investigation, Writing – original draft. LP: Visualization, Writing – review & editing. WY: Investigation, Resources, Supervision, Writing – review & editing. YL: Resources, Supervision, Validation, Writing – review & editing.
